# *Arthrospira maxima* Paradoxical Effect on *Trypanosoma cruzi* Infection

**Published:** 2020

**Authors:** Oscar A REBOREDA-HERNANDEZ, Adriana L. JUAREZ-SERRANO, Ivan GARCIA-LUNA, Nora L RIVERO-RAMIREZ, Rocio ORTIZ-BUTRON, Benjamín NOGUEDA-TORRES, Nayeli GONZALEZ-RODRIGUEZ

**Affiliations:** 1. Pathology Laboratory, Department of Morphology, Biological Sciences National School, National Polytechnic Institute, Mexico City, Mexico; 2. Neurobiology Laboratory, Department of Physiology, Biological Sciences National School, National Polytechnic Institute, Mexico City, Mexico; 3. Helminthology Laboratory, Department of Pathology, Biological Sciences National School, National Polytechnic Institute, Mexico City, Mexico

**Keywords:** Chagas disease, Dietary supplements, Spirulina, *Trypanosoma cruzi*

## Abstract

**Background:**

There are only two anti-trypanocidal drugs available, both have a lot of side effects. This is the pioneer study designed to evaluate the *Arthrospira maxima* effect in *Trypanosoma cruzi* -infected mice and macrophages.

**Methods:**

*A. maxima* was administered in vivo, and in vitro (120μL/mL; 200 μL/mL; 500 μL/mL; 852 μL/mL) as prophylaxis, and treatment. In vitro, phagocytosis and viability were measured in macrophages cultures supplemented with *A. maxima*, and *T. cruzi*-infected. In vivo *A. maxima* was supplemented to *T. cruzi*-infected mice in order to obtain the parasitemia curves, parasite amount, and histopathologic changes. This assay was performed in Biological Sciences National School of National Polytechnic Institute, Mexico City, in 2019.

**Results:**

In vivo, *A. maxima* administration exacerbates the immune innate host′s response, followed by mice early death. In vitro*, A. maxima* supplementation promote *T. cruzi*- macrophage phagocytosis, but also a sooner *T. cruzi*- infected macrophage death.

**Conclusion:**

*A. maxima* administration overactive the immune system, decreasing the parasitemia, but causing a severe tissue damage. Then, this nutraceutical has a paradoxical effect on intracellular parasitic infections such as Chagas disease.

## Introduction

Chagas’ disease, also known as American Trypanosomiasis, is a potentially life-threatening disease caused by the protozoan parasite, *Trypanosoma cruzi*. Currently, almost 8 million people are infected. This disease causes an average of 14,000 deaths per year. Its transmission is mostly vector-borne feces ingestion or autoinoculation, followed by vertical transmission, and organ transplant (1)

Chagas’s disease has 3 stages: acute, indeterminate, and chronic. In the acute stage the parasites are in the bloodstream, in the indeterminate stage the individual remains infected, but the parasite is barely bloodstream- founded. In the chronic stage the patients develop cardiomyopathy, heart failure and digestive-tract abnormalities such as megacolon, and megaesophagus. Diagnosis of chronic infection relies on serological testing through detection of immunoglobulin G (IgG) antibodies against *T. cruzi* (2–4).

There are only two anti-*T. cruzi* drugs in the health scheme of Chagas’ disease treatment: benznidazole, and nifurtimox. Furthermore, both drugs have low efficacy and numerous side effects that included anorexia, weight loss, neurological disorders, irritability, insomnia, disorientation, mood changes, paresthesia, seizures, and peripheral neuropathy, digestive manifestations such as nausea and vomiting, and occasionally, fever and rash. Besides, only a few people have access to these drugs (1%) (1,4–7).

On the other hand *Arthrospira maxima (A. maxima)* is a highly nutritional and ecofriendly nutraceutical cyanobacteria thoroughly investigated in the latest 30 years; for its role in human health management, and the US Food and Drug Administration (FDA) has categorized it as GRAS (Generally Recognized as Safe) (8–12).

*A. maxima* contains phycobiliproteins, allophycocyanin, and phycocyanin (PC), that inhibit the inducible nitric oxide synthase (iNOS), and cyclooxygeanase-2 (COX-2) (13, 14) that stimulates the production of antibodies, and up- or downregulates encoding cytokines genes (15,16), moreover, phycocyanobilin (PCB), inhibits the ONOO ^−^ mediated single-strand breaks in supercoiled plasmid DNA (17), allophycocyanin scavenge hydroxyl and peroxyl radicals, and bilirubin functions as a potent inhibitor of the reduced form of nicotinamide adenine dinucleotide phosphate (NADPH) oxidase activity (18). Moreover, *A. maxima* is considered as a powerful stimulator of the immune system by increasing the macrophages phagocytic activity, causing natural killer (NK) cells to accumulate in tissues, by stimulating the production of antibodies, and cytokines, and by activating and mobilizing T and B cells (16). All protective properties of *A. maxima* lead us to try this effect in a murine model infected by *T. cruzi*, etiological agent of American tripanosomiasis, a parasitosis whit a highly epidemically risk.

## Materials and Methods

In 2019*, Arthrospira maxima* fine powder (Alimentos Esenciales para la Humanidad, Mexico State, Mexico). *A. maxima* was dissolved in distilled water and sonicated by 5 pulses of 20 sec each, the lysis was observed using an optical microscope at 40X. All was performed by triplicate (19).

Animals: inbred BALB/c male mice were procured and maintained in temperature-controlled rooms (25±2 ºC) with access to water and food Rat Chow (Nestlé Purina, Vevey, Switzerland) *ad libitum* in the animal facilities of the Physiology Department, Escuela Nacional de Ciencias Biológicas (ENCB), Instituto Politécnico Nacional (IPN).

Mice were handled according the Animal Care and Use Committee Guide. The protocol for all experiments was evaluated and approved by the ENCB Bioethics Committee during 2017.

Parasite: *T. cruzi* NINOA strain (member of the predominant lineage in Mexico) (20) used in these experiments was kindly maintained and donated by PhD. B Nogueda-Torres (ENCB, IPN). Epimastigote forms were axenically cultured at 28 °C in 50 mL Liver Infusion Tryptose (LIT), the cells were counted every 24 h, using a Neubauer hemocytometer Blaubrand (Merck, Darmstadt, Germany) (19).

Macrophages: macrophages culture were obtained from peritoneal exudate BALB/c mice. Briefly, mononuclear cells separation was carried out by the density centrifugation method (Ficoll), cells were seeded in 9 culture flasks (triplicate) with Dulbecco Modified Eagles Minimal Essential Medium (DMEM) medium 25 mL Gibco BRL (Life Technologies, Grand Island, NY, USA) supplemented with 20% of fetal bovine serum (DMEM-FBS) Gibco BRL (Life Technologies, Grand Island, NY, USA), and incubated 60 min at 37 °C, and 5% CO _2_ atmosphere. The culture supernatant was aspirated to eliminate the non-adherent cells (lymphocytes and polymorphonuclear cells), then 12 mL DMEM medium were added (19). The most suitable confluence for each monolayer culture flask was 80% (21).

### Macrophages A. maxima phagocytosis in vitro assay

A macrophage culture was incubated 24h before the infection with 852 μg/mL (0.071 g/kg) *A. maxima* on the conditions mentioned above. This culture, and another four cultures with *A. maxima* at different concentrations 120 μg/mL (250 mg/kg), 200 μg/ml (500 mg/kg) and 500 μg/mL (1000 mg/kg) were infected with 1×10 ^3^ trypomastigotes. The parasite phagocytosis was counted every 2 h, for 6 h, using an inverted microscope CK2 (Olympus, Tokio, Japan) (100 cells were counted each time (for triplicate) (22).

### Parasitemia curves

Male mice were randomly divided into 8 groups of 6 mice each one: an uninfected group, a *T. cruzi*- parasitized group, 3 groups *A. maxima*-intragastrical daily treated at doses of 35.7, 71.4, and 142.8 mg/kg for prophylactic treatment two weeks before inoculation, and 3 groups treated with the same doses for post-infection as treatment.

To infect the mice 1×10 ^3^ metacyclic trypomastigotes were inoculated intraperitoneally. Blood parasite counting was done according to Brener method (Brener, 1962). Briefly, 50 μL of mouse peripheral blood obtained through a small cut in the caudal vein, then parasites were counted using a Neubauer camera. Mortality was recorded daily.

### Histopathologic Analyzes

Mice were sacrificed employing a CO _2_ camera at the maximum parasitemia peak according to the obtained curves, and heart was obtained. The organs were fixed with formaldehyde, paraffin included, cut in slices with a microtome at 5 μm, and each section was dyed with the hematoxylin- eosin (H-E) technique, dehydrated, and mounted in resin. The number of amastigote nests and other changes in the tissue under optical microscopy. Furthermore, the inflammatory infiltrate was ranked as it follows: absent (−), mild (<25% of the microscopic field), moderate (25%–50% of the microscopic field), and severe (>25% of the microscopic field).

### Statistical Analyzes

To analysis the parasitemia curves, Area under the curves was obtained, and ANOVA Two Ways Test was performed to correlate the groups’ data. Differences with *P*<0.05 were considered significant.

ANOVA test was applied to inflammation, and differences with *P*<0.05 (5%) were considered significant. After, non-parametric test Student-Newman-Keuls (SNK) were used, and results were expressed as mild, moderate and severe values. ANOVA test was also applied to correlate amastigotes nest amount, and differences with *P*<0.05 (5%) were considered significant. Moreover, to correlate inflammation and amastigotes amount, a post-hoc Principal Components Analysis (PCA) with a Pearson non-parametric rank test correlation was performed to correlate the histopathologic data.

To analyze mortality rates ANOVA Two Ways Test was performed to correlate the groups’ data. Differences with *P*<0.05 were considered significant. All tests were performed using GraphPad Prism 7® (GraphPad Software, La Jolla, CA, USA).

## Results

### T. cruzi macrophage phagocytosis

**Fig. 1:**
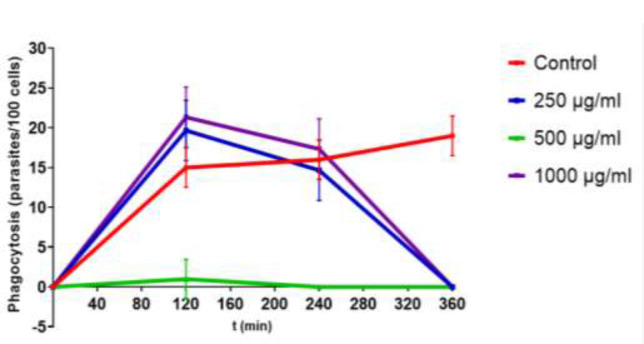
Phagocytosi= of *Trypanosoma cruzi*-infected macrophages in terms of parasites phagocyted for 100 cells, on medium supplemented with different *Arthrospira maxima* concentrations. The data is presented as mean ± S.D. of three cultures per group *P*-value: Infected controls vs. *A. maxima* 120 μg/mL; *A. maxima* 200 μg/mL; *A. maxima* 500 μg/mL (*P*<0.05)

Macrophages without *A. maxima* had a higher phagocytic activity (70%) that supplemented ones 852 μg/mL with *A. maxima* as prophylaxis (15%), and 120 μg/mL (21%), 200 μg/ml (2%) and 500 μg/mL (23%) as treatment (Fig. 1).

### Parasitemia curves

**Fig. 2:**
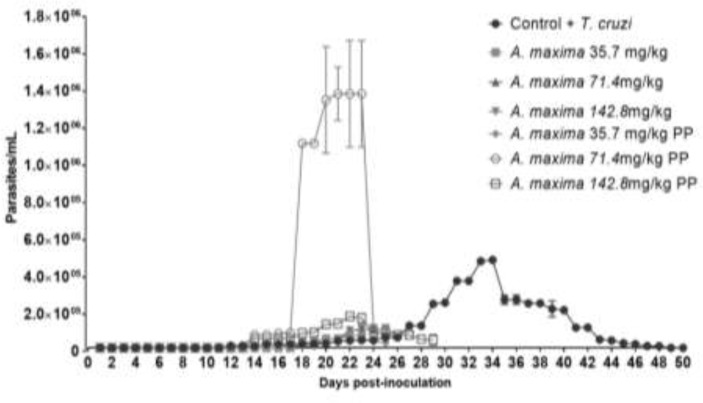
Parasitemia curves in terms of parasites/mL blood mice on the days post-inoculation, infected and supplemented with different *Arthrospira maxima* concentrations as treatment, and as prophylaxis (PP). The data is presented as mean ± S.D. of six mice per group: Infected controls vs. *A. maxima* 35.7 mg/kg; *A. maxima* 71.4 mg/kg; *A. maxima* 142.8 mg/kg as treatment; *A. maxima* 35.7 mg/kg; *A. maxima* 71.4 mg/kg; *A. maxima* 142.8 mg/kg as prophylaxis. *P*<0.05

The peak of the *T. cruzi*- infected group was recorded on day 34 post-infection (p.i.). Nevertheless, the concentration *A. maxima* administrated as prophylaxis groups has its parasitemia peak at day 25 p.i., and *A. maxima* (Fig. 2) administrated as treatment groups. All the treated groups showed significant reductions in parasitemia (*P*<0.05) when they were compared with control group.

### Parasitemia and mortality

**Fig. 3:**
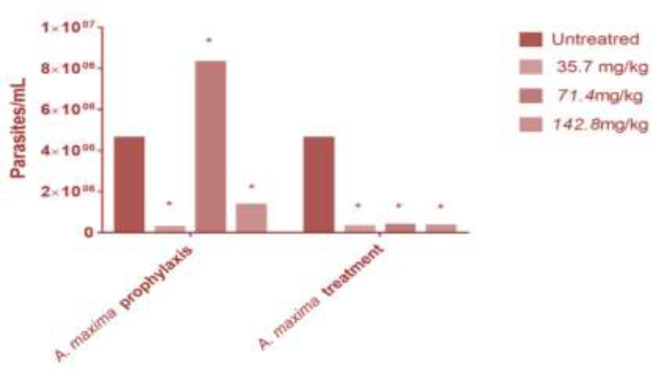
Parasitemia in terms of parasites/mL mice blood on the entire experiment, infected and supplemented with different *Arthrospira maxima* concentrations as treatment, and as prophylaxis (PP). The data is presented as mean ± S.D. of six mice per group: Infected controls vs. *A. maxima* 35.7 mg/kg; *A. maxima* 71.4 mg/kg; *A. maxima* 142.8 mg/kg as treatment; *A. maxima* 35.7 mg/kg; *A. maxima* 71.4 mg/kg; *A. maxima* 142.8 mg/kg as prophylaxis. *P*<0.05. The asterisk shows the difference between the untreated group and the supplemented groups

Infected mice parasitemia was 4.5×10 ^6^ parasites/mL, *A. maxima* administration lower the parasitemia 35.7 mg/kg (2.3×10 ^5^ parasites/mL), 71.4 mg/kg (8.2×10 ^6^ parasites/mL), and 142.8 mg/kg (1.3×10 ^6^ parasites/mL), as prophylaxis with, and 35.7 mg/kg (2.5×10 ^5^ parasites/mL), 71.4 mg/kg (3.0×10 ^5^ parasites/mL), and 142.8 mg/kg (2.6×10 ^5^ parasites/mL), as treatment (Fig. 3).

**Fig. 4:**
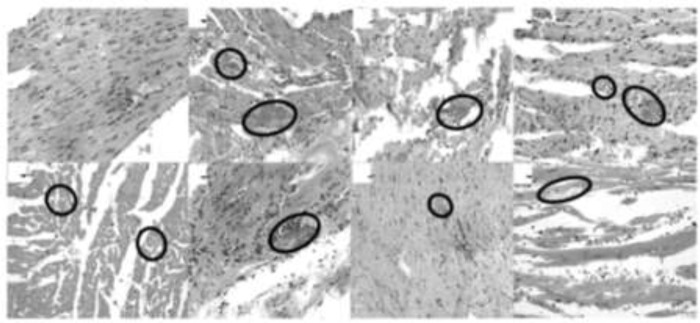
Histopathologic Analysis. Murine cardiac muscle infected with *T. cruzi* and supplemented with different concentrations of *A. maxima* as prophylaxis and as treatment. A) Cardiac tissue A) Healthy cardiac tissue B) *T. cruzi*- infected cardiac tissue C) *T. cruzi*- infected cardiac tissue treated with *A. maxima* 35.7 mg/kg as prophylaxis D) *T. cruzi*- infected cardiac tissue treated with *A. maxima* 71.4 mg/kg as prophylaxis E) *T. cruzi*- infected cardiac tissue treated with *A. maxima* 142.8 mg/kg as prophylaxis F) *T. cruzi*- infected cardiac tissue treated with *A. maxima* 35.7 mg/kg G) *T. cruzi*- infected cardiac tissue treated with *A. maxima* 71.4 mg/kg H) *T. cruzi*- infected cardiac tissue treated with *A. maxima* 142.8 mg/kg. 40X. Tec. H-E

Infected mice mortality rate was 60%. Mortality rises however *A. maxima* treatment (100%) was performed. It was presented sooner for the 35.7 mg/kg (day 26 p.i.), 71.4 mg/kg (day 28 p.i.), and 142.8 mg/kg (day 30 p.i.) prophylaxis with, and 35.7 mg/kg (day 23 p.i.), 71.4 mg/kg (day 25 p.i.), and 142.8 mg/kg (day 28 p.i.), as treatment than the control group (Fig. 4).

### Histopathology

**Fig. 5:**
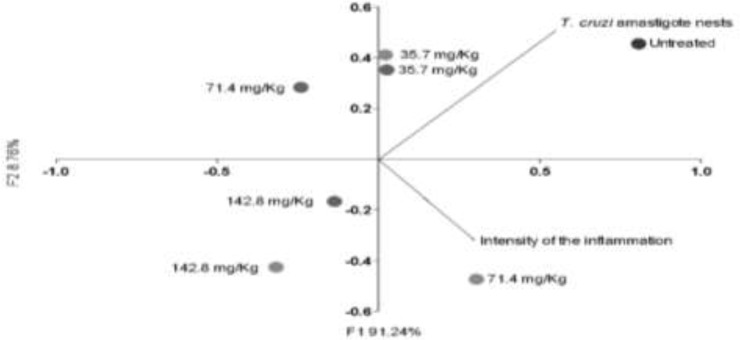
Histopathologic Analysis in terms of intensity of the inflammation, and *Trypanosoma cruzi* amastigote nests amount. The data is presented as mean ± S.D. of six mice per group *P*-value: Infected controls vs. *A. maxima* 35.7 mg/kg; *A. maxima* 71.4 mg/kg; *A. maxima* 142.8 mg/kg as treatment (gray dots); *A. maxima* 35.7 mg/kg; *A. maxima* 71.4 mg/kg; A. maxima 142.8 mg/kg as prophylaxis (black dots), comparison two ways ANOVA, *Post-hoc* analysis Pearson correlation and analyzed as an principal compounds analysis to correlate experimental groups with controls. *P*<0.05

Inflammatory infiltrate was more intense in the supplemented with *A. maxima* 71.4 mg/kg as treatment, and lower in the *A. maxima* 71.4mg/kg as prophylaxis. Higher amount of amastigote nest corresponds to the *T. cruzi*- infected *A. maxima*-untreated group, and the lower amastigote nests was reported for the *A. maxima* supplemented mice as treatment and the. Higher *A. maxima* dosage (142.8 mg/kg) as treatment has minor amount of nest but more inflammation, the same dosage administered as prophylaxis has milder inflammation but major amastigote nest amount. Lower *A. maxima* dosage as treatment and as prophylaxis behave similar (Figs. 4, 5).

## Discussion

Macrophages additionally to its phagocytic activity modulates cytokine production, interleukine-1 (IL-1), interleukine-6 (IL-6), and tumor necrosis factor (TNF-α), that derives in the anti-trypanocidal activity due to nitric oxide (NO) production (23).

*A. maxima* has a complex lipopolysaccharides cell wall that up-regulates the activity of the phagocytic macrophage, but these cells are the primary *T. cruzi*-niche (24). The main *A. maxima* compound phycocyanin leads to an immune response exacerbation due to the activation of extracellular signal-regulated kinases (ERK), c-Jun N-terminal kinases (JNK), p38 and IkB could lead to stress pathways activation (25). Besides *A. maxima* immuline rises macrophage activation through toll-like receptors 2 (TLR-2) receptors, nuclear factor kappa-light-chain-enhancer of activated B cells (NFkB) and leading to an interleukine-1β (IL-1β), and TNF-α production (26).

Moreover, reactive oxygen species (ROS) production overstimulation by the presence of *T. cruzi* itself (27) combined with the *A. maxima* stimuli lead to an excessive ROS augment that leads to macrophage lysis.

There are two macrophage phenotypes, macrophages phenotype 1 (M1) associated with the Th1 cytokine, and the rising iNOS, NO, ROS, interleukine-2 (IL-2), IL-1β than due to the microorganism death, but if the stimuli are constant could damage the tissue. Usually, macrophage phenotype 2 (M2) are involved in tissue remodeling, intracellular encapsulation, Th2 cytokines and upregulating interleukine-10 (IL-10) and transforming growth factor β (TGF-β) expression. In *T. cruzi*-infection the parasite leads to a M2 phenotype in order to leading to chronicity (28). Macrophages without *A. maxima* phagocyte a larger parasite amount in order to employ its cytoplasm to replicate itself due its antioxidant enzymes (29).

*Arthrospira* exerts an immunostimulatory activity through polysaccharides and glycolipids, which often serve as ligands of Toll receptors, mainly TLR-2 and TLR-4 (26). In the phagocytosis essays the macrophages were activated by the *A. maxima* lipopolysaccharide (LPS) (24), and they could induce NF-kB pathway, therefore increase both IL-1β and TNF-α (8)*,* and immuline (26). Our results suggest that this cyanobacterium upregulates the pro-inflammatory cytokines together with the pro-inflammatory anti *T. cruzi* inflammation itself lead to an exacerbated ROS release and could derivate on a tissue damage that exacerbate even more the innate immune response. According to this experiment, *A. maxima* treated-macrophages lysis occurs sooner that on control ones due a large amount of ROS generated, as was seen in another intracellular protozoa infections, with excessive ROS produces to mitochondrial dysfunction, followed by the caspases activation, and resulted on the cell apoptosis (30). The problem is that macrophage destruction leads *T. cruzi* (intracellular agent) to invade other cells.

Parasitemia on the *A. maxima* supplemented groups was lower than in the control due the high oxidative microenvironment generated as response of the parasite presence, and the inflammation upregulation generated by *A. maxima.* On the other hand, *A. maxima* contains provitamin A, and vitamin A modulates the IL1-β, IL-2, IL-6, and TNF-α cytokine production (16), but on the proved doses the stimuli were too strong, maybe it activates some stress pathways.

*Arthrospira platensis* modulates mitogen-activated protein kinases (MAPK) pathway (31), there are no information about *A. maxima* but as they are of the same genre, they could probably have an immunomodulatory effect employing the same stress pathway. Moreover, *T. cruzi* rises the inducible nitric oxide synthase (iNOS), and INF-gamma through activation of the NFkB, ERK1/2 MAPK stress pathway (32).

The excessive oxidative stress that is induced into the host because of the parasite presence and the cyanobacteria, exceeds the buffering capacity of the individual to defense its organs, resulting in cell damage and death, that could derivate on the failure of many organs, like the heart (32). *T. cruzi* cardiomyocytes invasion elicits the proinflammatory mediators (TNF-α, IL-1β, IFN-gamma, NO), increasing the amount of superoxide radical, hydrogen peroxyde, and peroxinitrite generation (33). NINOA-*T. cruzi* strain is cardiotropic, therefore, presence of the parasite is mostly on the heart, where the oxidative reaction could lead to the death of the mice, the higher the *A. maxima* concentration, the higher the oxidative stress that individuals suffered.

Mitochondrial stress suppressed the induced up-regulation in MYD88ASC, active caspase 1, and IL-1β in cardiomyocytes liberating mtDNA as a type of damage-associated molecular patterns (DAMPs) to stimulate inflammation through TLR-9 (31).

The uncontrolled macrophage-derived- oxidative burst elicit by *T. cruzi* by the generation of mitochondrial superoxide radicals leads to host cells to apoptosis (34). *Arthrospira* promotes cit C release adjuvate to caspase -3, and -9 activation by downregulation Bcl-2 (an anti-apoptotic protein), and upregulating Bax (pro-apoptotic protein) (31). Thus, as a result of the parasite infection, and the presence of *A. maxima* the M1 died before it has time to change its phenotype.

*Arthrospira* phycocyanin also induced autophagic cell death by activating p38 and JNK signaling while inhibiting Erk pathway (25), these biliproteins in macrophages LPS activated, inhibits the cyclooxygenase-2 (COX-2) leading to cell apoptosis fragmentation its DNA (13).

In the histopathological analyzes show that highest doses of *A. maxima* (142.8mg/kg) (regardless the administration scheme) have not a beneficial effect in *T. cruzi*-infected mice, the following doses (71.4 mg/kg) as treatment diminishes the inflammatory intensity as treatment (downregulate the pro-inflammatory activity) and as prophylaxis augment the intensity of the inflammation (upregulate the pro-inflammatory activity). The best results in this case were obtained with the minimum doses of *A. maxima* (35.7 mg/kg) however the administration scheme was (treatment or prophylaxis) because this dosage lows the parasite nest amount, and not increase the inflammation so much. The immune system is constantly activated by the cell apoptosis of the infected tissues, this apoptosis is related directly with the phycocyanin concentration (13).

However, as a pioneer experiment the employed doses were too immunostimulant, maybe by employing lower *A. maxima* doses the immunostimulatory activity could be beneficial for pathogens that do not take advantage of the macrophage activity. *A. maxima* has not only inflammatory but also anti-inflammatoryy activity by the ERK1/2, JNK y p38 pathways inhibition (12,16). This important issue remains as an interesting investigation subject.

## Conclusion

*Arthrospira maxima* supplementation in macrophages, promotes the phagocytosis, intensifies the ROS production leading them to death, decreasing the intracellular *T. cruzi* replicative niche, and therefore the parasitosis; however, the continuous exacerbation of the immune system, caused severe tissue damage that derived into individuals′ death.

Then, this nutraceutical has a paradoxical effect on intracellular parasitic infections such as Chagas′ disease.
